# Supplementary material for: Longitudinal Epidemiological Study of Autism Subgroups Using Autism Treatment Evaluation Checklist (ATEC) Score
Source: J Autism Dev Disord. 2018 Jul 30;50(5):1497–508. doi: 10.1007/s10803-018-3699-2 (PMC7211200; doi:10.1007/s10803-018-3699-2)
Supplement: Supplementary file 1 — Supplementary material 1 (DOCX 51 KB) [file 10803_2018_3699_MOESM1_ESM.docx]

# Supplementary Material

# Longitudinal Epidemiological Study of Autism Subgroups Using Autism Treatment Evaluation Checklist (ATEC) Score

Shreyas Mahapatra^1^, Edward Khokhlovich, Samantha Martinez^1^, Benjamin Kannel, Stephen M Edelson^2^, Andrey Vyshedskiy^1,3*^

^1^Boston University, Boston, USA

^2^Autism Research Institute, San Diego, CA, USA

^3^ImagiRation LLC, Boston, MA, USA

*Corresponding author: Andrey Vyshedskiy, Ph.D., Boston University, Boston, USA, Tel: +1 (617) 433-7724; E-mail: vysha@bu.edu

**Table S1**: Effect of sex on ATEC scores

| **Score Type** | **p-Value** |
| --- | --- |
| Total Score | 0.5495 |
| Subscale 1: Communication | 0.7033 |
| Subscale 2: Sociability | 0.7697 |
| Subscale 3: Sensory | 0.4942 |
| Subscale 4: Physical | 0.6388 |

**Table S2**: Effect of age on ATEC scores

| **Score Type** | **p-Value** |
| --- | --- |
| Total Score | <.0001 |
| Subscale 1: Communication | <.0001 |
| Subscale 2: Sociability | <.0001 |
| Subscale 3: Sensory | <.0001 |
| Subscale 4: Physical | 0.0469 |

**Table S3:** LS Means for ATEC total score in various age groups. Data are presented as LS Mean (SE; 95% CI) for age group data. The difference between Visit 8 and Visit 1 is presented as LS Mean (SE; P-value).

| **Visit Number** | **Age** | | |
| --- | --- | --- | --- |
|  | 2-3 YOA | 3-6 YOA | 6-12 YOA |
| Visit 1 | 64.41 (0.76: 62.93-65.90) | 62.15 (0.47: 61.24-63.07) | 61.98 (0.61; 60.78-63.17) |
| Visit 2 | 54.08 (0.91; 52.30-55.86) | 52.48 (0.56; 51.37-53.58) | 54.65 (0.77; 53.14-56.16) |
| Visit 3 | 50.07 (0.92; 48.26-51.88) | 49.97 (0.57; 48.85-51.08) | 52.78 (0.80; 51.22-54.35) |
| Visit 4 | 46.66 (0.95; 44.79-48.53) | 47.92 (0.59; 46.77-49.07) | 49.94 (0.79; 48.39-51.50) |
| Visit 5 | 43.09 (0.93; 41.25-44.92) | 45.15 (0.56; 44.06-46.24) | 49.02 (0.78; 47.49-50.54) |
| Visit 6 | 40.89 (0.99; 38.94-42.84) | 44.95 (0.62; 42.73-46.17) | 49.60 (0.81; 47.01-51.18) |
| Visit 7 | 38.93 (1.12; 36.73-41.14) | 44.49 (0.67; 43.18-45.80) | 49.08 (0.92; 47.28-50.87) |
| Visit 8 | 36.06 (1.28; 33.55-38.58) | 42.42 (0.72; 41.01-43.84) | 48.18 (0.95; 46.32-50.04) |
| Visit 8 – Visit 1 | -28.35 (1.30; <0.0001) | -19.73 (0.72; <0.0001) | -13.80 (0.96; <0.0001) |

**Table S4:** LS Means for Speech/Language/Communication subscale score for various age groups.

| **Visit Number** | **Age** | | |
| --- | --- | --- | --- |
|  | 2-3 YOA | 3-6 YOA | 6-12 YOA |
| Visit 1 | 15.58 (0.18; 15.23-15.93) | 15.12 (0.11; 14.90-15.33) | 14.84 (0.14; 14.56-15.12) |
| Visit 2 | 13.43 (0.21; 13.01-13.84) | 13.30 (0.13; 13.05-13.56) | 14.17 (0.18; 13.82-14.52) |
| Visit 3 | 12.08 (0.22; 11.66-12.50) | 12.66 (0.13; 12.41-12.92) | 13.62 (0.18; 13.26-13.98) |
| Visit 4 | 10.85 (0.22; 10.42-11.29) | 12.02 (0.13; 11.76-12.29) | 13.34 (0.18; 13.26-13.98) |
| Visit 5 | 9.60 (0.22; 9.17-10.03) | 11.59 (0.13; 11.34-11.85) | 13.29 (0.18; 12.94-13.64) |
| Visit 6 | 8.54 (0.23; 8.08-8.99) | 11.19 (0.14; 10.92-11.47) | 13.19 (0.19; 12.83-13.56) |
| Visit 7 | 7.91 (0.26; 7.41-8.42) | 10.99 (0.15; 10.69-11.29) | 13.26 (0.21; 12.84-13.67) |
| Visit 8 | 7.07 (0.29; 6.49-7.65) | 10.42 (0.16; 10.10-10.74) | 12.82 (0.22; 12.40-13.25) |
| Visit 8 – Visit 1 | -8.51 (0.29; <0.0001) | -4.70 (0.16; <0.0001) | -2.02 (0.22; <0.0001) |

**Table S5:** LS Means for Sociability subscale score for various age groups.

| **Visit Number** | **Age** | | |
| --- | --- | --- | --- |
|  | 2-3 YOA | 3-6 YOA | 6-12 YOA |
| Visit 1 | 13.84 (0.23; 13.39-14.28) | 13.11 (0.14; 12.84-13.39) | 13.27 (0.18; 12.90-13.63) |
| Visit 2 | 10.81 (0.28; 10.26-11.35) | 10.66 (0.17; 10.32-11.00) | 11.24 (0.24; 10.77-11.71) |
| Visit 3 | 9.83 (0.28; 9.27-10.39) | 10.34 (0.17; 10.00-10.68) | 11.08 (0.25; 10.59-11.56) |
| Visit 4 | 9.24 (0.29; 8.66-9.82) | 9.99 (0.18; 9.63-10.34) | 9.87 (0.25; 9.39-10.35) |
| Visit 5 | 8.60 (0.29; 8.04-9.17) | 9.23 (0.17; 8.89-9.56) | 10.03 (0.24; 9.56-10.50) |
| Visit 6 | 8.13 (0.31; 7.53-8.74) | 9.65 (0.19; 9.28-10.03) | 10.05 (0.25; 9.55-10.54) |
| Visit 7 | 7.92 (0.35; 7.23-8.61) | 9.26 (0.21; 8.86-9.67) | 10.06 (0.29; 9.49-10.62) |
| Visit 8 | 6.92 (0.40; 6.12-7.71) | 8.87 (0.23; 8.42-9.31) | 10.05 (0.30; 9.47-10.65 |
| Visit 8 – Visit 1 | -6.92 (0.42; <0.0001) | -4.25 (0.23; <0.0001) | -3.21 (0.31; <0.0001) |

**Table S6:** LS Means for Sensory/Cognitive awareness subscale score for various age groups.

| **Visit Number** | **Age** | | |
| --- | --- | --- | --- |
|  | 2-3 YOA | 3-6 YOA | 6-12 YOA |
| Visit 1 | 14.80 (0.22; 14.37-15.22) | 14.26 (0.13; 13.99-14.52) | 14.31 (0.18; 13.96-14.65) |
| Visit 2 | 12.12 (0.26; 11.60-12.63) | 12.30 (0.16; 11.98-12.62) | 12.82 (0.22; 12.38-13.26) |
| Visit 3 | 11.12 (0.27; 10.60-11.65) | 11.56 (0.16; 11.24-11.88) | 11.87 (0.23; 11.41-12.32) |
| Visit 4 | 10.14 (0.28; 9.60-10.68) | 11.14 (0.17; 10.80-11.47) | 11.53 (0.23; 11.08-11.98) |
| Visit 5 | 9.57 (0.27; 9.04-10.10) | 10.35 (0.16; 10.03-10.66) | 11.17 (0.23; 10.74-11.63) |
| Visit 6 | 9.29 (0.29; 8.73-9.86) | 10.32 (0.18; 9.97-10.67) | 11.18 (0.24; 10.72-11.65) |
| Visit 7 | 8.39 (0.33; 7.75-9.03) | 10.49 (0.19; 10.11-10.87) | 10.96 (0.27; 10.43-11.48) |
| Visit 8 | 8.45 (0.37; 7.71-9.19) | 9.75 (0.21; 9.33-10.16) | 10.65 (0.28; 10.10-11.19) |
| Visit 8 – Visit 1 | -6.35 (0.38; <0.0001) | -4.51 (0.21; <0.0001) | -3.66 (0.28; <0.0001) |

**Table S7:** LS Means for Physical/Motor Behavior subscale score for various age groups.

| **Visit Number** | **Age** | | |
| --- | --- | --- | --- |
|  | 2-3 YOA | 3-6 YOA | 6-12 YOA |
| Visit 1 | 19.94 (0.34; 19.28-20.60) | 20.10 (0.21; 19.70-20.51) | 20.61 (0.28; 20.07-21.15) |
| Visit 2 | 17.40 (0.41; 16.60-18.21) | 16.48 (0.26; 15.97-16.98) | 17.33 (0.35; 16.63-18.02) |
| Visit 3 | 16.57 (0.42; 15.75-17.39) | 15.69 (0.26; 15.19-16.20) | 17.09 (0.37; 16.37-17.81) |
| Visit 4 | 16.07 (0.43; 15.22-16.92) | 15.06 (0.27; 14.53-15.58) | 16.12 (0.36; 15.40-16.83) |
| Visit 5 | 14.89 (0.42; 14.06-15.73) | 14.25 (0.25; 13.76-14.75) | 15.45 (0.36; 14.75-16.15) |
| Visit 6 | 14.56 (0.45; 13.67-15.45) | 14.12 (0.28; 13.56-14.68) | 16.13 (0.37; 15.40-16.87) |
| Visit 7 | 13.07 (0.52; 13.29-15.32) | 14.05 (0.31; 13.45-14.65) | 15.70 (0.42; 14.87-16.53) |
| Visit 8 | 13.26 (0.59; 12.09-14.42) | 13.67 (0.33; 13.02-14.33) | 15.59 (0.44; 14.73-16.45) |
| Visit 8 – Visit 1 | -8.51 (0.20; <0.0001) | -4.70 (0.16; <0.0001) | -2.02 (0.22; <0.0001) |

**Table S8:** LS Mean Differences in ATEC total score between age groups. Data are presented as LS Mean difference (SE; P-Value)

| **Visit Number** | **Age Groups** | | |
| --- | --- | --- | --- |
|  | 2-3 vs. 3-6 | 2-3 vs. 6-12 | 3-6 vs. 6-12 |
| Visit 1 | -1.23 (2.72; 1.0000) | 2.44 (0.93; 0.7182) | 0.17 (0.71; 1.0000) |
| Visit 2 | 1.60 (1.02; 0.9998) | -0.57 (1.16; 1.0000) | -2.18 (0.91; 0.8730) |
| Visit 3 | 0.10 (1.04; 1.0000 | -2.71 (1.19; 0.9167) | -2.82 (0.94; 0.4059) |
| Visit 4 | 1.39 (3.56; 1.0000) | 0.13 (3.49; 1.0000) | -1.89 (3.52; 1.0000) |
| Visit 5 | -2.07 (1.04; 0.9862) | -5.93 (1.18; 0.0002) | -3.86 (0.91; 0.0090) |
| Visit 6 | -4.06 (1.13; 0.0918) | -8.71 (1.25; <0.0001) | -4.65 (0.98; 0.0009) |
| Visit 7 | -5.56 (1.27; 0.0053) | -10.1 (1.42; <0.0001) | -4.58 (1.10; 0.0116) |
| Visit 8 | -6.36 (1.44; 0.0042) | -12.12 (1.57; <0.0001) | -5.76 (1.16; 0.0003) |

**Table S9:** LS Mean Differences in Speech/Language/Communication subscale score between Age Groups.

| **Visit Number** | **Age Groups** | | |
| --- | --- | --- | --- |
|  | 2-3 vs. 3-6 | 2-3 vs. 6-12 | 3-6 vs. 6-12 |
| Visit 1 | 0.46 (0.20; 0.8958) | 0.74 (0.23; 0.2288) | 0.27 (0.17; 0.9994) |
| Visit 2 | 0.12 (0.24; 1.0000) | -0.74 (0.27; 0.6665) | -0.86 (0.21; 0.0176) |
| Visit 3 | -0.58 (0.24; 0.8653) | -1.54 (0.28; <0.0001) | -0.95 (0.22; 0.0045) |
| Visit 4 | -1.17 (0.25; 0.0014) | -2.48 (0.28; <0.0001) | -1.31 (0.22; <0.0001) |
| Visit 5 | -2.00 (0.24; <0.0001) | -3.69 (0.28; <0.0001) | -1.69 (0.21; <0.0001) |
| Visit 6 | -2.66 (0.26; <0.0001) | -4.66 (0.29; <0.0001) | -2.00 (0.23; <0.0001) |
| Visit 7 | -3.08 (0.29; <0.0001) | -5.34 (0.33; <0.0001) | -2.26 (0.25; <0.0001) |
| Visit 8 | -3.35 (0.33; <0.0001) | -5.75 (0.36; <0.0001) | -2.40 (0.27; <0.0001) |

**Table S10:** LS Mean Differences in Sociability subscale score between Age Groups.

| **Visit Number** | **Age Groups** | | |
| --- | --- | --- | --- |
|  | 2-3 vs. 3-6 | 2-3 vs. 6-12 | 3-6 vs. 6-12 |
| Visit 1 | 0.72 (0.25; 0.5291) | 0.57 (0.28; 0.9810) | -0.15 (0.22; 1.0000) |
| Visit 2 | 0.15 (0.32; 1.0000) | -0.43 (0.36; 1.000) | -0.58 (0.28; 0.9764) |
| Visit 3 | -0.51 (0.32; 0.9997) | -1.24 (0.37; 0.1750) | -0.74 (0.29; 0.7879) |
| Visit 4 | -0.75 (0.33; 0.9358) | -0.63 (0.37; 0.9990) | 0.12 (0.29; 1.0000) |
| Visit 5 | -0.62 (0.32; 0.9911) | -1.43 (09.37; 0.0336) | -0.80 (0.28; 0.5450) |
| Visit 6 | -1.52 (0.35; 0.0067) | -1.92 (0.39; 0.0004) | -0.40 (0.30; 1.0000) |
| Visit 7 | -1.34 (0.40; 0.1859) | -2.13 (0.45; 0.0009) | -0.79 (0.34; 0.9128) |
| Visit 8 | -1.95 (0.45; 0.0072) | -3.14 (0.49; <0.0001) | -1.18 (0.37; 0.2465) |

**Table S11:** LS Mean Differences in Sensory/Cognitive awareness subscale score between Age Groups.

| **Visit Number** | **Age Groups** | | |
| --- | --- | --- | --- |
|  | 2-3 vs. 3-6 | 2-3 vs. 6-12 | 3-6 vs. 6-12 |
| Visit 1 | 0.54 (0.24; 0.9327) | 0.49 (0.27; 0.9963) | -0.05 (0.21; 1.0000) |
| Visit 2 | -0.18 (0.30; 1.0000) | -0.71 (0.34; 0.9711) | -0.52 (0.27; 0.9873) |
| Visit 3 | -0.44 (0.30; 1.0000) | -0.74 (0.35; 0.9595) | -0.31 (0.27 1.0000) |
| Visit 4 | -1.00 (0.13; 0.2874) | -1.39 (0.35; 0.0270) | -0.39 (0.27; 1.0000) |
| Visit 5 | -0.78 (0.30; 0.7657) | -1.61 (0.34; 0.0012) | -0.83 (0.27; 0.3109 |
| Visit 6 | -1.03 (0.33; 0.3299) | -1.89 (0.36; 0.0001) | -0.87 (0.29; 0.3920) |
| Visit 7 | -2.10 (0.37; <0.0001) | -2.57 (0.42; <0.0001) | -0.47 (0.32; 0.9999) |
| Visit 8 | -1.30 (0.42; 0.3549) | -2.20 (0.46; 0.0008) | -0.90 (0.34; 0.7028) |

**Table S12:** LS Mean Differences in Physical/Motor Behavior subscale score between Age Groups.

| **Visit Number** | **Age Groups** | | |
| --- | --- | --- | --- |
|  | 2-3 vs. 3-6 | 2-3 vs. 6-12 | 3-6 vs. 6-12 |
| Visit 1 | 0.46 (0.64; 1.0000) | 0.74 (0.23; 0.2288) | 0.27 (0.17; 0.9994) |
| Visit 2 | 0.12 (0.24; 1.0000) | -0.74 (0.27; 0.6665) | -0.86 (0.21; 0.0176) |
| Visit 3 | -0.58 (0.24; 0.8653) | -1.54 (0.28; <0.0001) | -0.95 (0.22; 0.0045) |
| Visit 4 | -1.17 (0.25; 0.0014) | -2.48 (0.28; <0.0001) | -1.31 (0.22; <0.0001) |
| Visit 5 | -2.00 (0.24; <0.0001) | -3.69 (0.28; <0.0001) | -1.68 (0.21; <0.0001) |
| Visit 6 | -2.66 (0.26; <0.0001) | -4.66 (0.29; <0.0001) | -2.00 (0.23; <0.0001) |
| Visit 7 | -3.08 (0.29; <0.0001) | -5.34 (0.33; <0.0001) | -2.26 (0.25; <0.0001) |
| Visit 8 | -3.35 (0.33; <0.0001) | -5.75 (0.36; <0.0001) | -2.40 (0.27; <0.0001) |

**Table S13:** Effects of a participants’ country of origin (English-speaking countries group and non-English-speaking countries group) on ATEC scores

| **Score Type** | **p-Value** |
| --- | --- |
| Total Score | <0.0001 |
| Subscale 1: Speech/Language/Communication | <0.0001 |
| Subscale 2: Sociability | <0.0001 |
| Subscale 3: Sensory/Cognitive Awareness | 0.0484 |
| Subscale 4: Health/Physical/Behavior | 0.0040 |

**Table S14:** LS Means for ATEC total score of English-Speaking and non-English-speaking countries. Data are presented as LS Mean (SE; 95% CI) for individual severity groups. The difference between Visit 8 and Visit 1 is presented as LS Mean (SE; P-value).

| **Visit Number** | **Non-English-speaking** | **English Speaking** |
| --- | --- | --- |
| Visit 1 | 62.92 (0.67; 61.61-64.23) | 62.09 (0.69; 60.74-63.44) |
| Visit 2 | 53.21 (0.74; 51.77-54.65) | 53.83 (0.78; 52.30-55.35) |
| Visit 3 | 50.09 (0.74; 48.64-51.54) | 51.87 (0.79; 50.32-53.42) |
| Visit 4 | 47.35 (0.75; 45.88-48.81) | 49.64 (0.80; 48.06-51.21) |
| Visit 5 | 44.95 (0.73; 43.51-46.38) | 47.24 (0.78; 45.71-48.77) |
| Visit 6 | 44.29 (0.76; 42.80-45.77) | 47.25 (0.84; 45.61-48.89) |
| Visit 7 | 43.25 (0.81; 41.66-48.84) | 47.08 (0.89; 45.34-48.83) |
| Visit 8 | 41.34 (0.86; 39.66-43.03) | 45.40 (0.93; 43.58-47.21) |
| Visit 8 – Visit 1 | -21.58 (0.70; <0.0001) | -16.70 (0.80; <0.0001) |

**Table S15:** LS Means for Speech/Language/Communication subscale score of English-Speaking and non-English-speaking countries.

| **Visit Number** | **Non-English-speaking** | **English Speaking** |
| --- | --- | --- |
| Visit 1 | 15.54 (0.16; 15.23-15.86) | 14.98 (0.16; 14.66-15.30) |
| Visit 2 | 13.89 (0.18; 13.54-14.23) | 13.66 (0.18; 13.29-14.02) |
| Visit 3 | 13.08 (0.18; 12.74-13.43) | 12.97 (0.19; 12.60-13.34) |
| Visit 4 | 12.27 (0.18; 11.92-12.62) | 12.55 (0.19; 12.17-12.92) |
| Visit 5 | 11.78 (0.17; 11.44-12.13) | 12.05 (0.19; 11.69-12.42) |
| Visit 6 | 11.34 (0.18; 10.98-11.69) | 11.67 (0.20; 11.28-12.06) |
| Visit 7 | 10.93 (0.19; 10.55-11.31) | 11.81 (0.21; 11.40-12.23) |
| Visit 8 | 10.54 (0.20; 10.14-10.93) | 11.09 (0.22; 10.66-11.52) |
| Visit 8 – Visit 1 | -5.01 (0.16; <0.0001) | -3.89 (0.19; <0.0001) |

**Table S16:** LS Means for Sociability subscale score of English-Speaking and non-English-speaking countries.

| **Visit Number** | **Non-English-speaking** | **English Speaking** |
| --- | --- | --- |
| Visit 1 | 13.42 (0.19; 13.04-13.81) | 13.29 (0.20; 12.89-13.69) |
| Visit 2 | 10.81 (0.22; 10.38-11.24) | 11.14 (0.23; 10.68-11.59) |
| Visit 3 | 10.20 (0.22; 9.77-10.63) | 10.99 (0.24; 10.53-11.46) |
| Visit 4 | 9.52 (0.22; 9.09-9.96) | 10.44 (0.24; 9.97-10.92) |
| Visit 5 | 9.17 (0.22; 8.74-9.60) | 9.75 (0.23; 9.29-10.21) |
| Visit 6 | 9.12 (0.22; 8.68-9.56) | 10.12 (0.25; 9.63-10.61) |
| Visit 7 | 8.87 (0.24; 8.39-9.34) | 9.94 (0.27; 9.41-10.47) |
| Visit 8 | 8.35 (0.26; 7.84-8.86) | 9.73 (0.28; 9.17-10.28) |
| Visit 8 – Visit 1 | -5.07 (0.22; <0.0001) | -3.57 (0.26; <0.0001) |

**Table S17:** LS Means for Sensory/Cognitive awareness score of English-Speaking and non-English-speaking countries.

| **Visit Number** | **Non-English-speaking** | **English-speaking** |
| --- | --- | --- |
| Visit 1 | 14.33 (0.19; 13.96-14.70) | 14.31 (0.20; 13.92-14.69) |
| Visit 2 | 12.18 (0.21; 11.77-12.59) | 12.59 (0.22; 12.15-13.03) |
| Visit 3 | 11.25 (0.21; 10.83-11.66) | 1.90 (0.23; 11.45-12.35) |
| Visit 4 | 10.79 (0.21; 10.37-11.21) | 11.31 (0.23; 10.85-11.76) |
| Visit 5 | 10.22 (0.21; 9.81-10.63) | 10.61 (0.22; 10.17-11.05) |
| Visit 6 | 10.11 (0.22; 9.69-10.54) | 10.62 (0.24; 10.15-11.09) |
| Visit 7 | 9.98 (0.23; 9.53-10.44) | 10.49 (0.26; 9.98-10.99) |
| Visit 8 | 9.35 (0.25; 8.86-9.83) | 10.24 (0.27; 9.71-10.76) |
| Visit 8 – Visit 1 | -4.98 (0.21; <0.0001) | -4.07 (0.24; <0.0001) |

**Table S18:** LS Means for Health/Physical/Behavior subscale score of English-Speaking and non-English-speaking countries.

| **Visit Number** | **Non-English-speaking** | **English-speaking** |
| --- | --- | --- |
| Visit 1 | 20.06 (0.29; 19.49-20.63) | 20.37 (0.30; 19.78-20.97) |
| Visit 2 | 16.60 (0.32; 15.96-17.24) | 17.20 (0.35; 16.52-17.88) |
| Visit 3 | 15.80 (0.33; 15.16-16.44) | 16.73 (0.35; 16.03-17.42) |
| Visit 4 | 15.03 (0.33; 14.39-15.68) | 16.09 (0.36; 15.38-16.79) |
| Visit 5 | 14.04 (0.32; 13.40-14.67) | 15.55 (0.35; 14.87-16.23) |
| Visit 6 | 14.04 (0.33; 13.39-14.70) | 15.61 (0.37; 14.87-16.34) |
| Visit 7 | 13.80 (0.36; 13.09-14.51) | 15.51 (0.40; 14.72-16.30) |
| Visit 8 | 13.42 (0.38; 12.68-14.19) | 15.02 (0.42; 14.20-15.84) |
| Visit 8 – Visit 1 | -6.63 (0.33; <0.0001) | -5.35 (0.38; <0.0001) |

**Table S19:** LS Mean Differences in ATEC total score between English-Speaking and non-English-speaking countries. Data are presented as LS Mean difference (SE; P-Value)

| **Visit Number** | **Non-English-speaking vs. English-speaking** |
| --- | --- |
| Visit 1 | 0.83 (0.61; 0.9937) |
| Visit 2 | -0.62 (0.77; 1.0000) |
| Visit 3 | -1.77 (0.79; 0.6677) |
| Visit 4 | -2.29 (0.81; 0.2526) |
| Visit 5 | -2.29 (0.76; 0.1862) |
| Visit 6 | -3.00 (0.85; 0.0423) |
| Visit 7 | -3.83 (0.95; 0.0056) |
| Visit 8 | -4.05 (1.02; 0.0056) |

**Table S20:** LS Mean Differences in Speech/Language/Communication subscale score between English-Speaking and non-English-speaking countries.

| **Visit Number** | **Non-English-speaking vs. English-speaking** |
| --- | --- |
| Visit 1 | 0.56 (0.15; 0.0115) |
| Visit 2 | 0.23 (0.18; 0.9973) |
| Visit 3 | 0.11 (0.19; 1.0000) |
| Visit 4 | -0.28 (0.19; 0.9878) |
| Visit 5 | -0.27 (0.18; 0.9856) |
| Visit 6 | -0.33 (0.20; 0.9570) |
| Visit 7 | -0.88 (0.22; 0.0085) |
| Visit 8 | -0.55 (0.24; 0.6330) |

**Table S21:** LS Mean Differences in Sociability subscale score between English-Speaking and non-English-speaking countries.

| **Visit Number** | **Non-English-speaking vs. English-speaking** |
| --- | --- |
| Visit 1 | 0.13 (0.19; 1.0000) |
| Visit 2 | -0.32 (0.24; 0.9941) |
| Visit 3 | -0.79 (0.25; 0.0922) |
| Visit 4 | -0.92 (0.25; 0.0237) |
| Visit 5 | -0.58 (0.24; 0.5356) |
| Visit 6 | -1.00 (0.27; 0.0172) |
| Visit 7 | -1.08 (0.30; 0.0283) |
| Visit 8 | -1.37 (0.32; 0.0023) |

**Table S22:** LS Mean Differences in Sensory/Cognitive Awareness subscale score between English-Speaking and non-English-speaking countries.

| **Visit Number** | **Non-English-speaking vs. English-speaking** |
| --- | --- |
| Visit 1 | 0.02 (0.18; 1.0000) |
| Visit 2 | -0.41 (0.23; 0.9131) |
| Visit 3 | -0.65 (0.23; 0.2494) |
| Visit 4 | -0.51 (0.24; 0.7110) |
| Visit 5 | -0.39 (0.23; 0.9382) |
| Visit 6 | -0.51 (0.25; 0.8053) |
| Visit 7 | -0.50 (0.28; 0.9119) |
| Visit 8 | -0.89 (0.30; 0.1845) |

**Table S23:** LS Mean Differences in Health/Physical/Behavior subscale score between English-Speaking and non-English-speaking countries.

| **Visit Number** | **Non-English-speaking vs. English-speaking** |
| --- | --- |
| Visit 1 | -0.32 (0.28; 0.9990) |
| Visit 2 | -0.60 (0.35; 0.9504) |
| Visit 3 | -0.93 (0.36; 0.4259) |
| Visit 4 | -1.05 (0.37; 0.2466) |
| Visit 5 | -1.51 (0.35; 0.0021) |
| Visit 6 | -1.56; 0.39; 0.0065) |
| Visit 7 | -1.71 (0.44; 0.0098) |
| Visit 8 | -1.60 (0.47; 0.0619) |

**Table S24:** Effects of initial ASD severity on ATEC scores

| **Score Type** | **p-Value** |
| --- | --- |
| Total Score | <0.0001 |
| Subscale 1: Communication | <0.0001 |
| Subscale 2: Sociability | <0.0001 |
| Subscale 3: Sensory | <0.0001 |
| Subscale 4: Physical | <0.0001 |

**Table S25:** LS Means for ATEC total score for various severity groups. In Tables S17 to S26 the severity group was assigned based solely on a child’s initial ATEC total score (independent of child’s age). Data are presented as LS Mean (SE; 95% CI) for individual severity groups. The difference between Visit 8 and Visit 1 is presented as LS Mean (SE; P-value).

| **Visit Number** | **Severity** | | |
| --- | --- | --- | --- |
|  | Mild | Moderate | Severe |
| Visit 1 | 56.44 (0.92; 54.64-58.25) | 62.94 (0.69; 61.60-64.29) | 70.57 (1.10; 68.40-72.73) |
| Visit 2 | 52.93 (1.00; 50.98-54.89) | 54.11 (0.77; 52.60-55.63) | 53.47 (1.19; 51.14-55.81) |
| Visit 3 | 51.26 (1.01; 49.28-53.23) | 51.13 (0.77; 49.61-52.64) | 49.92 (1.22; 47.53-52.31) |
| Visit 4 | 48.57 (1.01; 46.58-50.56) | 48.39 (0.79; 46.85-49.94) | 48.17 (1.22; 45.78-50.57) |
| Visit 5 | 47.72 (1.01; 45.74-49.69) | 45.75 (0.77; 44.24-47.26) | 44.30 (1.19; 41.96-44.64) |
| Visit 6 | 46.46 (1.04; 44.42-48.51) | 45.80 (0.80; 44.23-47.37) | 43.81 (1.25; 41.36-46.26) |
| Visit 7 | 47.12 (1.08; 45.00-49.25) | 44.99 (0.87; 43.29-46.69) | 40.97 (1.34; 38.35-43.59) |
| Visit 8 | 45.25 (1.13; 43.02-47.47) | 42.38 (0.90; 40.61-44.16) | 41.04 (1.41; 38.28-43.81) |
| Visit 8 – Visit 1 | -11.20 (0.87; <0.0001) | -20.56 (0.76; <0.0001) | -29.52 (1.10; <0.0001) |

**Table S26:** LS Means for Speech/Language/Communication subscale score for various severity groups.

| **Visit Number** | **Severity** | | |
| --- | --- | --- | --- |
|  | Mild | Moderate | Severe |
| Visit 1 | 14.62 (0.19; 14.25-14.99) | 15.41 (0.17; 15.09-15.74) | 15.94 (0.20; 15.55-16.33) |
| Visit 2 | 13.58 (0.21; 13.17-14.00) | 13.91 (0.19; 13.54-14.27) | 13.76 (0.23; 13.31-14.21) |
| Visit 3 | 13.00 (0.21;12.58-13.42) | 13.04 (0.19; 12.68-13.41) | 12.94 (90.23; 12.48-13.41) |
| Visit 4 | 12.21 (0.22; 11.79-12.64) | 12.26 (0.19; 11.89-12.63) | 12.76 (0.24; 12.30-13.22) |
| Visit 5 | 11.93 (0.21; 11.51-12.35) | 11.72 (0.19; 11.35-12.08) | 12.09 (0.23; 11.64-12.53) |
| Visit 6 | 11.57 (0.22; 11.13-12.01) | 11.22 (0.19; 10.84-11.60) | 11.70 (0.24; 11.22-12.18) |
| Visit 7 | 11.62 (0.23; 11.16-12.08) | 11.23 (0.21; 10.82-11.64) | 10.74 (0.27; 10.21-11.27) |
| Visit 8 | 10.96 (0.25; 10.49-11.46) | 10.49 (0.22; 10.07-10.92) | 10.89 (0.29; 10.31-11.46) |
| Visit 8 – Visit 1 | -3.64 (0.21; <0.0001) | -4.92 (0.18; <0.0001) | -5.05 (0.27; <0.0001) |

**Table S27:** LS Means for Sociability subscale score for various severity groups.

| **Visit Number** | **Severity** | | |
| --- | --- | --- | --- |
|  | Mild | Moderate | Severe |
| Visit 1 | 10.93 (0.23; 10.48-11.39) | 13.44 (0.20; 13.05-13.84) | 16.39 (0.26; 15.88-16.91) |
| Visit 2 | 10.28 (0.27; 9.76-10.79) | 11.02 (0.23; 10.57-11.48) | 11.60 (0.30; 11.01-12.19) |
| Visit 3 | 10.19 (0.27; 9.67-10.72) | 10.53 (0.23; 10.08-10.98) | 10.79 (0.31; 10.17-11.40) |
| Visit 4 | 9.46 (0.27; 8.93-9.99) | 9.86 (0.24; 9.39-10.32) | 10.46 (0.31; 9.85-11.07) |
| Visit 5 | 9.46 (0.27; 9.84-9.99) | 9.27 (0.23; 8.81-9.72) | 9.55 (0.30; 8.96-10.13) |
| Visit 6 | 9.15 (0.28; 8.94-9.99) | 9.27 (0.23; 8.81-9.72) | 9.55 (0.30; 8.96-10.13) |
| Visit 7 | 9.31 (0.30; 8.73-9.89) | 9.09 (0.26; 8.56-9.61) | 9.40 (0.36; 8.70-10.10) |
| Visit 8 | 9.07 (0.31; 8.45-9.69) | 8.61 (0.28; 8.07-9.15) | 9.01 (0.39; 8.25-9.76) |
| Visit 8 – Visit 1 | -1.87 (0.28; <0.0001) | -4.83 (0.25; <0.0001) | -7.39 (0.36; <0.0001) |

**Table S28:** LS Means for Sensory/Cognitive awareness subscale score for various severity groups.

| **Visit Number** | **Severity** | | |
| --- | --- | --- | --- |
|  | Mild | Moderate | Severe |
| Visit 1 | 12.70 (0.23; 12.26-13.15) | 14.48 (0.20; 14.09-14.87) | 16.22 (0.25; 15.72-16.72) |
| Visit 2 | 12.00 (0.26; 11.50-12.50) | 12.45 (0.22; 12.01-12.89) | 12.66 (0.29; 12.10-13.23) |
| Visit 3 | 11.42 (0.26; 10.91-11.93) | 11.58 (0.22; 11.14-12.02) | 11.50 (0.30; 10.91-12.08) |
| Visit 4 | 10.85 (0.26; 10.34-11.37) | 10.97 (0.23; 10.52-11.42) | 11.28 (0.30; 10.70-11.87) |
| Visit 5 | 10.66 (0.26; 10.15-11.17) | 10.23 (0.22; 9.86-10.73) | 10.21 (0.29; 9.65-10.77) |
| Visit 6 | 10.46 (0.27; 9.92-10.99) | 10.23 (0.23; 9.77-10.68) | 10.29 (0.31; 9.68-10.89) |
| Visit 7 | 10.65 (0.29; 10.09-11.21) | 10.20 (0.25; 9.971-10.70) | 9.36 (0.34; 8.69-10.03) |
| Visit 8 | 10.09 (0.30; 9.50-10.68) | 9.59 (0.26; 9.07-10.11) | 9.30 (0.37; 8.58-10.02) |
| Visit 8 – Visit 1 | -2.62 (0.26; <0.0001) | -4.89 (0.23; <0.0001) | -6.92 (0.33; <0.0001) |

**Table S29:** LS Means for Health/Physical/Behavior subscale score for various severity groups.

| **Visit Number** | **Severity** | | |
| --- | --- | --- | --- |
|  | Mild | Moderate | Severe |
| Visit 1 | 17.51 (0.34; 16.85-18.17) | 19.95 (0.30; 19.36-20.54) | 24.26 (0.39; 23.50-25.02) |
| Visit 2 | 16.30 (0.38; 15.55-17.05) | 16.92 (0.35; 16.24-17.59) | 17.50 (0.44; 16.63-18.37) |
| Visit 3 | 15.83 (0.39; 15.06-16.60) | 16.14 (0.34; 15.47-16.82) | 16.70 (0.46; 15.80-17.59) |
| Visit 4 | 15.22 (0.39; 14.44-15.99) | 15.50 (0.35; 14.71-16.19) | 15.75 (0.46; 14.85-16.65) |
| Visit 5 | 14.86 (0.39; 14.10-15.62) | 14.67 (0.39; 14.10-15.62) | 14.47 (0.44; 13.60-15.33) |
| Visit 6 | 14.53 (0.41; 13.72-15.33) | 14.97 (0.36; 14.27-15.67) | 14.27 (0.48; 13.34-15.20) |
| Visit 7 | 14.76 (0.43;13.91-15.61) | 14.66 (0.39; 13.89-15.43) | 15.58 (0.53; 12.55-14.61) |
| Visit 8 | 14.36 (0.46; 13.45-15.26) | 13.89 (0.41; 13.09-14.69) | 13.89 (0.57; 12.78-15.01) |
| Visit 8 – Visit 1 | -3.15 (0.41; <0.0001) | -6.07 (0.36; <0.0001) | -10.37 (0.52; <0.0001) |

**Table S30:** LS Mean Differences in ATEC total score between various severity groups. Data are presented as LS Mean difference (SE; P-Value)

| **Visit Number** | **Severity Groups** | | |
| --- | --- | --- | --- |
|  | Mild vs. Moderate | Mild vs. Severe | Moderate vs. Severe |
| Visit 1 | -6.50 (0.91; <0.0001) | -14.12 (1.55; <0.0001) | -7.62 (1.05; <0.0001) |
| Visit 2 | -1.18 (1.05; 1.0000) | -0.54 (1.65; 1.0000) | 0.64 (1.20; 1.0000) |
| Visit 3 | 0.13 (1.06; 1.0000) | 1.33 (1.68; 1.0000) | 1.20 (1.22; 1.0000) |
| Visit 4 | 0.17 (1.08; 1.0000) | 0.39 (1.68; 1.0000) | 0.22 (1.24; 1.0000) |
| Visit 5 | 2.00 (1.06; 0.9756) | 3.42 (1.66; 0.9291) | 1.45 (1.19; 1.0000) |
| Visit 6 | 0.66 (1.12; 1.0000) | 2.65 (1.73; 0.9980 | 1.99 (1.28; 0.9976) |
| Visit 7 | 2.13 (1.20; 0.9854) | 6.15 (1.81; 0.1059) | 4.02 (1.40; 0.3815) |
| Visit 8 | 2.86 (1.27; 0.8449) | 4.20 (1.89; 0.8604) | 1.34 (1.49; 1.0000) |

**Table S31:** LS Mean Differences in Speech/Language/Communication subscale score between various severity groups.

| **Visit Number** | **Severity Groups** | | |
| --- | --- | --- | --- |
|  | Mild vs. Moderate | Mild vs. Severe | Moderate vs. Severe |
| Visit 1 | -2.51 (0.23; <0.0001) | -5.46 (0.31; <0.0001) | -2.95 (0.26; <0.0001) |
| Visit 2 | -0.75 (0.28; 0.5712) | -1.33 (0.37; 0.0586) | -0.58 (0.32; 0.9814) |
| Visit 3 | -0.33 (0.29; 1.0000 | -0.59 (0.38; 0.9976) | -0.26 (0.33; 1.0000) |
| Visit 4 | -0.39 (0.29; 0.9998) | -1.00 (0.38; 0.5884) | -0.61 (0.33; 0.9794) |
| Visit 5 | 0.20 (0.29; 1.0000) | -0.08 (0.37; 1.0000) | -0.30 (0.31; 1.0000) |
| Visit 6 | -0.47 (0.31; 0.9984) | -0.55 (0.40; 0.9997) | -0.08 (0.35; 1.0000) |
| Visit 7 | -0.21 (0.34; 1.0000) | -0.09 (0.44; 1.0000) | -0.30 (0.39; 1.0000) |
| Visit 8 | 0.46 (0.37; 0.9999) | 0.06 (0.47; 1.0000) | -0.39 (0.43; 1.0000) |

**Table S32:** LS Mean Differences in Sociability subscale score between various severity groups.

| **Visit Number** | **Severity Groups** | | |
| --- | --- | --- | --- |
|  | Mild vs. Moderate | Mild vs. Severe | Moderate vs. Severe |
| Visit 1 | -1.78 (0.22; <0.0001) | -3.51 (0.31; <0.0001) | -1.74 (0.24; <0.0001) |
| Visit 2 | -0.45 (0.27; 0.9949) | -0.66 (0.35; 0.9753) | -0.21 (0.30; 1.0000) |
| Visit 3 | -0.16 (0.28; 1.0000) | -0.08 (0.37; 1.0000) | -0.08 (0.31; 1.0000) |
| Visit 4 | -0.12 (0.28; 1.0000) | -0.43 (0.37; 1.0000) | -0.31 (0.31; 1.0000) |
| Visit 5 | -0.37 (0.27; 0.9998) | -0.45 (0.36; 0.9999) | -0.09 (0.30; 1.0000) |
| Visit 6 | -0.23 (0.30; 1.0000) | 0.17 (0.38; 1.0000) | -0.06 (0.37; 0.8215) |
| Visit 7 | 0.45 (0.32; 0.9996) | 1.20 (0.42; 0.2414) | 0.84 (0.37; 0.8215) |
| Visit 8 | -0.50 (0.35; 0.9993) | 0.79 (0.45; 0.9887) | 0.29 (0.40; 1.0000) |

**Table S33:** LS Mean Differences in Sensory/Cognitive awareness subscale score between various severity groups.

| **Visit Number** | **Severity Groups** | | |
| --- | --- | --- | --- |
|  | Mild vs. Moderate | Mild vs. Severe | Moderate vs. Severe |
| Visit 1 | -2.45 (0.33; <0.0001) | -6.75 (0.44; <0.0001) | -4.31 (0.38; <0.0001) |
| Visit 2 | -0.62 (0.41; 0.9984) | -1.20 (0.52; 0.8207) | -0.58 (0.47; 0.9999) |
| Visit 3 | -0.31 (0.41; 1.0000) | -0.87 (0.54; 0.9966) | -0.55 (0.48; 1.0000) |
| Visit 4 | -0.29 (0.42; 1.0000) | -0.54 (0.55; 1.0000) | -0.25 (0.49; 1.0000) |
| Visit 5 | 0.19 (0.41; 1.0000) | 0.39 (0.53; 1.0000) | 0.20 (0.46; 1.0000) |
| Visit 6 | -0.44 (0.44; 1.0000) | 0.26 (0.57; 1.0000) | 0.70 (0.51; 0.9997) |
| Visit 7 | 0.10 (0.49; 1.0000) | 1.18 (0.63; 0.9722) | 1.08 (0.58; 0.9734) |
| Visit 8 | 0.46 (0.53; 1.0000) | 0.46 (0.68; 1.0000) | -0.01 (0.63; 1.0000) |

**Table S34:** LS Mean Differences in Health/Physical/Behavior subscale score between various severity groups.

| **Visit Number** | **Severity Groups** | | |
| --- | --- | --- | --- |
|  | Mild vs. Moderate | Mild vs. Severe | Moderate vs. Severe |
| Visit 1 | -5.24 (0.84; <0.0001) | -8.57 (1.17; <0.0001) | -3.33 (0.89; 0.0366) |
| Visit 2 | 0.12 (1.01; 1.0000) | -0.88(1.30; 1.0000) | -1.00 (1.07; 1.0000) |
| Visit 3 | -1.26 (1.02; 0.9999) | -2.02 (1.32; 0.9981) | -0.75 (1.08; 1.0000) |
| Visit 4 | -2.38 (1.05; 0.8294) | -2.26 (1.32; 0.9918) | 0.13 (1.09; 1.0000) |
| Visit 5 | -0.67 (1.02; 1.0000) | -1.81 (1.31; 0.9996) | -1.14 (1.06; 1.0000) |
| Visit 6 | -1.20 (1.10; 1.0000) | -4.62 (1.36; 0.1100) | -3.42 (1.13; 0.2723) |
| Visit 7 | 0.86 (1.20; 1.0000) | -1.72 (1.47; 1.0000) | -2.58 (1.23; 0.9164) |
| Visit 8 | -0.43 (1.31; 1.0000) | -4.55 (1.52; 0.2999) | -4.12 (1.33; 0.2341) |

**Table S35:** Effects of severity groups on ATEC scores. In Tables S31 to S41 the severity group was assigned based on a child’s initial ATEC total score and age as defined in Table 11.

| **Score Type** | **p-Value** |
| --- | --- |
| Total Score | <0.0001 |
| Subscale 1: Communication | <0.0001 |
| Subscale 2: Sociability | <0.0001 |
| Subscale 3: Sensory | <0.0001 |
| Subscale 4: Physical | <0.0001 |

**Table S36:** LS Means for ATEC total score for various severity groups. Data are presented as LS Mean (SE; 95% CI) for individual severity groups. The difference between Visit 8 and Visit 1 is presented as LS Mean (SE; P-value).

| **Visit Number** | **Severity** | | |
| --- | --- | --- | --- |
|  | Mild | Moderate | Severe |
| Visit 1 | 57.58 (0.90; 55.80-59.35) | 62.82 (0.75; 61.35-64.29) | 66.15 (0.81; 64.55-67.74) |
| Visit 2 | 52.91 (0.98; 50.98-54.84) | 52.79 (0.85; 51.13-54.45) | 53.79 (0.92; 51.99-55.59) |
| Visit 3 | 49.43 (1.00; 47.47-51.39) | 50.69 (0.85; 49.02-52.36) | 51.45 (0.93; 49.63-53.27) |
| Visit 4 | 46.45 (1.01; 44.48-48.43) | 48.84 (0.87; 47.13-50.54) | 48.71 (0.93; 46.88-50.54) |
| Visit 5 | 44.82 (1.00; 42.86-46.78) | 45.48 (0.84; 43.83-47.13) | 46.62 (0.91; 44.83-48.42) |
| Visit 6 | 43.29 (1.04; 41.24-44.33) | 44.48 (0.89; 42.73-46.23) | 47.90 (0.94; 41.62-45.31) |
| Visit 7 | 44.33 (1.11; 42.16-46.50) | 43.47 (0.94; 41.62-45.31) | 46.05 (1.03; 44.02-48.08) |
| Visit 8 | 41.12 (1.15; 38.87-43.36) | 41.55 (103; 39.52-43.57) | 45.67 (1.06; 43.58-47.75) |
| Visit 8 – Visit 1 | -16.46 (0.92; <0.0001) | -21.27 (0.90; <0.0001) | -20.48 (0.90; <0.0001) |

**Table S37:** LS Means for Speech/Language/Communication subscale score for various severity groups.

| **Visit Number** | **Severity** | | |
| --- | --- | --- | --- |
|  | Mild | Moderate | Severe |
| Visit 1 | 15.22 (0.20; 14.83-15.60) | 15.41 (0.18; 15.06-15.76) | 14.94 (0.18; 14.60-15.29) |
| Visit 2 | 13.71 (0.22; 13.28-14.13) | 14.70 (0.20; 13.31-14.10) | 13.57 (0.20; 13.17-13.96) |
| Visit 3 | 12.67 (0.22; 12.24-13.10) | 13.04 (0.20; 12.64-13.43) | 13.03 (0.20; 12.63-13.43) |
| Visit 4 | 11.52 (0.22; 11.08-11.95) | 12.48 (0.21; 12.07-12.88) | 12.78 (0.21; 12.38-13.19) |
| Visit 5 | 10.98 (0.22; 10.55-11.41) | 11.74 (0.20; 11.35-12.14) | 12.61 (0.20; 12-21-13.00) |
| Visit 6 | 10.30 (0.23; 9.85-10.76) | 11.08 (0.21; 10.66-11.49) | 12.63 (0.21; 12.22-13.04) |
| Visit 7 | 10.46 (0.25; 9.98-10.94) | 12.00 (0.22; 10.56-11.43) | 12.10 (0.23; 11.65-12.56) |
| Visit 8 | 9.42 (0.26; 8.92-9.92) | 10.60 (0.24; 10.12-11.08) | 11.90 (0.24; 11.44-12.37) |
| Visit 8 – Visit 1 | -5.80 (0.21; <0.0001) | -4.81 (0.21; <0.0001) | -3.04 (0.21; <0.0001) |

**Table S38:** LS Means for Sociability subscale score for various severity groups.

| **Visit Number** | **Severity** | | |
| --- | --- | --- | --- |
|  | Mild | Moderate | Severe |
| Visit 1 | 11.34 (0.25; 10.85-11.82) | 13.35 (0.22; 12.92-13.78) | 14.73 (0.23; 14.29-15.18) |
| Visit 2 | 10.40 (0.28; 9.86-10.95) | 10.48 (0.25; 9.99-10.98) | 11.40 (0.27; 10.88-11.92) |
| Visit 3 | 9.82 (0.28; 9.27-10.37) | 10.24 (0.26; 9.74-10.74) | 10.94 (0.27; 10.42-11.47) |
| Visit 4 | 9.23 (0.29; 8.67-9.79) | 9.83 (0.27; 9.32-10.35) | 10.08 (0.27; 9.55-10.61) |
| Visit 5 | 8.93 (0.28; 83.8-9.48) | 9.06 (0.25; 8.56-9.55) | 9.73 (0.26; 9.22-10.25) |
| Visit 6 | 8.65 (0.30; 8.07-9.24) | 9.36 (0.27; 8.83-9.88) | 10.01 (0.27; 9.48-10.55) |
| Visit 7 | 8.88 (0.32; 8.25-9.51) | 8.75 (0.29; 7.66-8.91) | 9.79 (0.31; 9.19-10.39) |
| Visit 8 | 8.32 (0.33; 7.66-8.97) | 8.28 (0.32; 7.66-8.91) | 9.65 (0.32; 9.03-10.27) |
| Visit 8 – Visit 1 | -3.02 (0.30; <0.0001) | -5.07 (0.29; <0.0001) | -5.09 (0.29; <0.0001) |

**Table S39:** LS Means for Sensory/Cognitive awareness subscale score for various severity groups.

| **Visit Number** | **Severity** | | |
| --- | --- | --- | --- |
|  | Mild | Moderate | Severe |
| Visit 1 | 12.97 (0.24; 12.50-13.45) | 14.37 (0.21; 13.95-14.79) | 15.19 (0.22; 14.76-15.62) |
| Visit 2 | 11.92 (0.27; 11.39-12.44) | 12.08 (0.24; 11.61-12.57) | 12.70 (0.25; 12.21-13.20) |
| Visit 3 | 10 94 (0.27; 10.40-11.47) | 11.47 (0.24; 10.99-11.95) | 11.77 (0.26; 11.27-12.28) |
| Visit 4 | 10.31 (0.28; 9.77-10.85) | 10.97 (0.25; 10.48-11.46) | 11.37 (0.26; 10.87-11.88) |
| Visit 5 | 9.98 (0.27; 9.45-10.51) | 10.12 (0.24; 9.64-10.59) | 10.73 (0.25; 10.23-11.22) |
| Visit 6 | 9.82 (0.29; 9.25-10.38) | 9.84 (0.26; 9.34-10.35) | 10.98 (0.26; 10.47-11.50) |
| Visit 7 | 9.92 (0.31; 9.32-10.52) | 9.87 (0.27; 9.34-10.40) | 10.48 (0.29; 9.90-11.05) |
| Visit 8 | 9.23 (0.32; 8.61-9.86) | 9.30 (0.30; 8.71-9.89) | 10.29 (0.30; 9.71-10.88) |
| Visit 8 – Visit 1 | -3.74 (0.27; <0.0001) | -5.07 (0.27; <0.0001) | -4.90 (0.26; <0.0001) |

**Table S40:** LS Means for Health/Physical/Behavior subscale score for various severity groups.

| **Visit Number** | **Severity** | | |
| --- | --- | --- | --- |
|  | Mild | Moderate | Severe |
| Visit 1 | 17.47 (0.36; 16.76-18.18) | 19.86 (0.33; 19.22-20.50) | 22.52 (0.34; 21.87-23.18) |
| Visit 2 | 16.23 (0.40; 15.44-17.02) | 16.53 (0.38; 15.79-17.26) | 17.20 (0.39; 16.44-17.97) |
| Visit 3 | 15.27 (0.41; 14.46-16.08) | 15.97 (0.38; 15.22-16.71) | 16.71 (0.40; 15.94-17.49) |
| Visit 4 | 14.66 (0.42; 13.85-15.48) | 15.59 (0.40; 14.83-16.35) | 15.57 (0.40; 14.79-16.36) |
| Visit 5 | 14.20 (0.41; 13.39-15.00) | 14.62 (0.37; 13.89-15.35) | 14.62 (0.39; 13.86-15.38) |
| Visit 6 | 13.86 (0.44; 13.00-14.71) | 14.23 (0.40; 13.45-15.01) | 15.42 (0.40; 14.63-16.22) |
| Visit 7 | 14.36 (0.47; 13.44-15.27) | 13.90 (0.42; 13.07-14.73) | 14.75 (0.45; 13.87-15.63) |
| Visit 8 | 13.50 (0.49; 12.53-14.45) | 13.31 (0.47; 12.42-14.25) | 14.92 (0.46; 14.01-15.83) |
| Visit 8 – Visit 1 | -3.97 (0.43; <0.0001) | -6.53 (0.42; <0.0001) | -7.60 (0.42; <0.0001) |

**Table S41:** LS Mean Differences in ATEC total score between Severity Groups. Data are presented as LS Mean difference (SE; P-Value).

| **Visit Number** | **Severity Groups** | | |
| --- | --- | --- | --- |
|  | Mild vs. Moderate | Mild vs. Severe | Moderate vs. Severe |
| Visit 1 | -5.24 (0.84; <0.0001) | -8.57 (1.17; <0.0001) | -3.33 (0.89; 0.0366) |
| Visit 2 | 0.12 (1.01; 1.0000) | -0.88 (1.30; 1.0000) | -1.00 (1.07; 1.0000) |
| Visit 3 | -1.26 (1.02; 0.9999) | -2.02 (1.32; 0.9981) | -0.75 (1.08; 1.0000) |
| Visit 4 | -2.38 (1.05; 0.8294) | -2.26 (1.33; 0.9918) | 0.13 (1.09; 1.0000) |
| Visit 5 | -0.67 (1.02; 1.0000) | -1.81 (1.31; 0.9996) | -1.14 (1.06; 1.000) |
| Visit 6 | -1.20 (1.10; 1.0000) | -4.62 (1.36; 0.1100) | -3.42 (1.13; 0.2723) |
| Visit 7 | 0.86 (1.20; 1.0000) | -1.72 (1.47; 1.0000) | -2.58 (1.23; 0.9164) |
| Visit 8 | -0.42 (1.31; 1.0000) | -4.55 (1.52; 0.2999) | -0.43 (1.31; 1.0000) |

**Table S42:** LS Mean Differences in Speech/Language/Communication subscale score between Severity Groups.

| **Visit Number** | **Severity Groups** | | |
| --- | --- | --- | --- |
|  | Mild vs. Moderate | Mild vs. Severe | Moderate vs. Severe |
| Visit 1 | -0.19 (0.18; 1.0000) | 0.27 (0.22; 0.9999) | 0.47 (0.19; 0.7170) |
| Visit 2 | 0.01 (0.22; 1.0000) | 0.14 (0.25; 1.0000) | 3.10 (0.26; <0.0001) |
| Visit 3 | -0.36 (0.23; 0.9965) | -0.36 (0.26; 0.9996) | -0.01 (0.24; 1.0000) |
| Visit 4 | -0.96 (0.23; 0.0090) | -1.27 (0.26; 0.0003) | -0.31 (0.24; 0.9999) |
| Visit 5 | -0.76 (0.22; 0.1086) | -1.62 (0.26; <0.0001) | -0.86 (0.23; 0.0369) |
| Visit 6 | -0.77 (0.25; 0.2124) | -2.33 (0.27; <0.0001) | -1.55 (0.25; <0.0001) |
| Visit 7 | -0.54 (0.27; 0.9474) | -1.64 (0.30; <0.0001) | -1.11 (0.28; 0.0129) |
| Visit 8 | -1.18 (0.30; 0.0144) | -2.48 (0.31; <0.00001) | -1.30 (0.30; 0.0029) |

**Table S43:** LS Mean Differences in Sociability subscale score between Severity Groups.

| **Visit Number** | **Severity Groups** | | |
| --- | --- | --- | --- |
|  | Mild vs. Moderate | Mild vs. Severe | Moderate vs. Severe |
| Visit 1 | -2.01 (0.24; <0.0001) | -3.39 (0.30; <0.00010 | -1.38 (0.25; <0.0001) |
| Visit 2 | -0.08 (0.30; 1.0000) | -1.00 (0.35; 0.3942) | -0.92 (0.31; 0.3263) |
| Visit 3 | -0.42 (0.30; 0.9996) | -1.12 (0.36; 0.2073) | -0.70 (0.31; 0.8602) |
| Visit 4 | -0.61 (0.31; 0.9612) | -0.86 (0.36; 0.7642) | -0.25 (0.32; 1.0000) |
| Visit 5 | -0.13 (0.30; 1.0000) | -0.80 (0.35; 0.8302) | -0.67 (0.31; 0.8749) |
| Visit 6 | -0.70 (09.33; 0.9041) | -1.36 (0.37; 0.0506) | -0.66 (0.33; 0.9526) |
| Visit 7 | 0.13 (0.36; 1.0000) | -0.91 (0.41; 0.8761) | -1.03 (0.37; 0.4405) |
| Visit 8 | 0.03 (0.40; 1.0000) | -1.33 (0.42; 0.2493) | -1.36 (0.40; 0.1106) |

**Table S44:** LS Mean Differences in Sensory/Cognitive awareness subscale score between Severity Groups.

| **Visit Number** | **Severity Groups** | | |
| --- | --- | --- | --- |
|  | Mild vs. Moderate | Mild vs. Severe | Moderate vs. Severe |
| Visit 1 | -1.40 (0.23; <0.0001) | -2.22 (0.29; <0.0001) | -0.82 (0.24; 0.0928) |
| Visit 2 | -0.17 (0.28; 1.0000) | -0.79 (0.34; 0.7855) | -0.62 (0.29; 0.9107) |
| Visit 3 | -0.53 (0.29; 0.9783) | -0.83 (0.34; 0.7219) | -0.30 (0.30; 1.0000) |
| Visit 4 | -0.65 (0.30; 0.8642) | -1.06 (0.34; 0.2468) | -0.41 (0.30; 0.9998) |
| Visit 5 | -0.14 (0.28; 1.0000) | -0.75 (0.34; 0.8660) | -0.61 (0.29; 0.9210) |
| Visit 6 | -0.02 (0.31; 1.0000) | -1.17 (0.36; 0.1529) | -1.14 (0.31; 0.0488) |
| Visit 7 | 0.04 (0.34; 1.0000) | -0.56 (0.39; 0.9994) | -0.60 (0.35; 0.9894) |
| Visit 8 | -0.07 (0.38; 1.0000) | -1.06 (0.41; 0.6066) | -0.99 (0.38; 0.5689) |

**Table S45:** LS Mean Differences in Health/Physical/Behavior subscale score between Severity Groups.

| **Visit Number** | **Severity Groups** | | |
| --- | --- | --- | --- |
|  | Mild vs. Moderate | Mild vs. Severe | Moderate vs. Severe |
| Visit 1 | -2.39 (0.35; <0.0001) | -5.06 (0.43; <0.0001) | -2.66 (0.37; <0.0001) |
| Visit 2 | -0.29 (0.43; 1.0000) | -0.97 (0.50; 0.9646) | -0.68 (0.46; 0.9989) |
| Visit 3 | -0.70 (0.44; 0.9971) | -1.44 (0.51 (0.4312) | -0.75 (0.46; 0.9961) |
| Visit 4 | -0.93 (0.45; 0.9307) | -0.91 (0.52; 0.9884) | -0.02 (0.48; 1.0000) |
| Visit 5 | -0.42 (0.44; 1.0000) | -0.42 (0.51; 1.0000) | -0.00 (0.45; 1.0000) |
| Visit 6 | -0.38 (0.48; 1.0000) | -1.57 (0.54; 0.3647) | -1.19 (0.49; 0.7226) |
| Visit 7 | 0.45 (0.48; 1.0000) | -0.39 (0.60; 1.0000) | -0.85 (0.54; 0.9975) |
| Visit 8 | 0.17 (0.58; 1.0000) | -1.42; 0.63; 0.8286) | -1.59 (0.59; 0.5206 |
